# Classification of Various Marijuana Varieties by Raman Microscopy and Chemometrics

**DOI:** 10.3390/toxics10030115

**Published:** 2022-02-28

**Authors:** Luis Ramos-Guerrero, Gemma Montalvo, Marzia Cosmi, Carmen García-Ruiz, Fernando E. Ortega-Ojeda

**Affiliations:** 1 Centro de Investigación de Alimentos, CIAL—Centro de Investigación de Alimentos, Universidad UTE, Quito EC170527, Ecuador; luis.ramos@ute.edu.ec; 2 Universidad de Alcalá, Departamento de Química Analítica, Química Física e Ingeniería Química, Ctra. Madrid–Barcelona km 33,600, 28871 Alcalá de Henares, Madrid, Spain; carmen.gruiz@uah.es; 3 Universidad de Alcalá, Instituto Universitario de Investigación en Ciencias Policiales (IUICP), Calle Libreros 27, 28801 Alcalá de Henares, Madrid, Spain; 4 Department of Engineering and Architecture, University of Trieste Via Alfonso Valerio 6a, 34127 Trieste, Italy; marzia.cosmi92@gmail.com; 5 Universidad de Alcalá, Departamento de Ciencias de la Computación, Ctra. Madrid–Barcelona km 33,600, 28871 Alcalá de Henares, Madrid, Spain

**Keywords:** marijuana, trichome, chemometrics, Raman microscopy, discrimination, OPLS-DA

## Abstract

The Raman analysis of marijuana is challenging because of the sample’s easy photo-degradation caused by the laser intensity. In this study, optimization of collection parameters and laser focusing on marijuana trichome heads allowed collecting Raman spectra without damaging the samples. The Raman spectra of Δ^9^-tetrahydrocannabinol (THC), cannabidiol (CBD), and cannabinol (CBN) standard cannabinoids were compared with Raman spectra of five different types of marijuana: four Sativa varieties (Amnesia Haze, Amnesia Hy-Pro, Original Amnesia, and Y Griega) and one Indica variety (Black Domina). The results verified the presence of several common spectral bands that are useful for marijuana characterization. Results were corroborated by the quantum chemical simulated Raman spectra of their acid-form (tetrahydrocannabinol acid (THCA), cannabidiol acid (CBDA)) and decarboxylated cannabinoids (THC, CBD, and CBN). A chemometrics-assisted method based on Raman microscopy and OPLS-DA offered good classification among the different marijuana varieties allowing identification of the most significant spectral bands.

## 1. Introduction

*Cannabis sativa* L. is an annual, dioecious herb, belonging to the genus of flowering plants in the Cannabaceae family and originating from Eastern and Central Asia. It has been employed from ancient times as a source of a stem fiber (hemp) and a resinous intoxicant (marijuana). Hemp has been used as source of textiles, as an edible plant, and as a medicinal and psychoactive plant. At present, hemp fibers are used to produce bioplastic and antibacterial agents among other biotechnological applications [1]. For drug use, the interest regarding this plant is due to the unique compounds that it can produce: the phytocannabinoids [2]. Phytocannabinoids, when they are consumed, act on the central nervous system (CNS) and peripheral nervous system (PNS), causing an alteration of the perception, producing euphoric, analgesic, and other effects [3,4]. These effects explain the wide cultivation and consumption of cannabis-derived products nowadays. 

**Figure 1:**
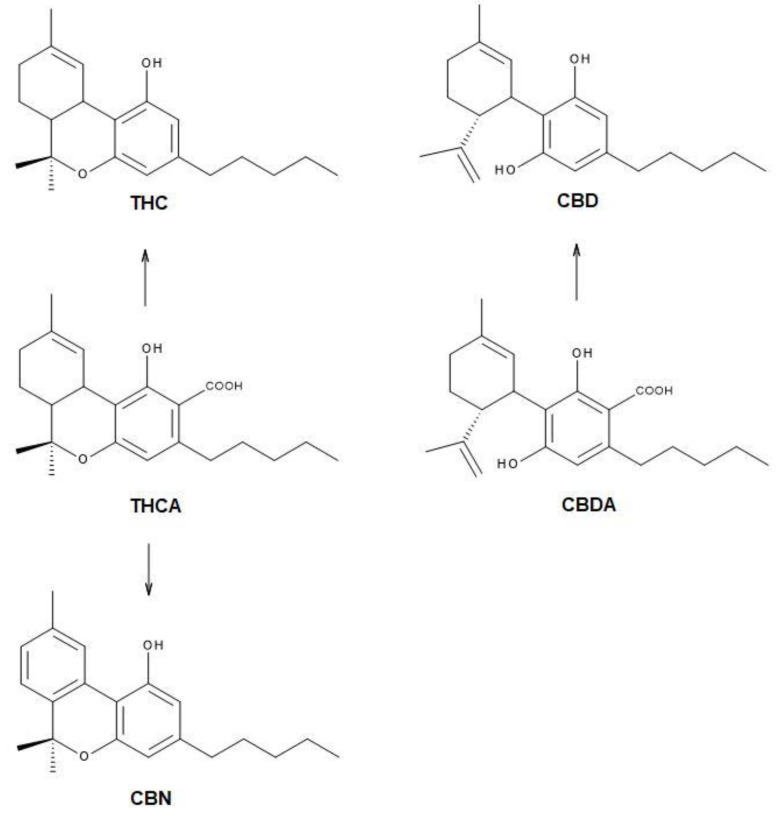
Molecular structure of tetrahydrocannabinol acid (THCA) and cannabidiol acid (CBDA). In the presence of heat or light, they decompose to decarboxylate molecules: Δ^9^-tetrahydrocannabinol (THC) or cannabidiol (CBD). The cannabinol (CBN) is an indicator of the thermal decomposition of cannabinoids, through prolonged exposure to elevated temperatures.

Marijuana refers to the dried leaves, flowers, stems, and seeds from the cannabis plant. After alcohol, it is the most commonly used psychotropic drug in the United States [5]. In Europe, cannabis has a prevalence of use about five times larger than other substances, and it is the most widely used illicit drug available [6]. Whether hemp or drug, *Cannabis* has only one genus with only one species (Sativa) that is highly variable. Nevertheless, Sativa can also be classified in *C. sativa* variety *Sativa*, *C. sativa* variety *Indica, C. sativa* variety *Ruderalis*, and *C. sativa* variety *Afghanica* [1]. Several metabolites called cannabinoids have been reported for those plants. The acid form of those compounds is predominant in the fresh tissues; for example, the most common and concentrated compounds are tetrahydrocannabinol acid (THCA) and cannabidiol acid (CBDA). When acid cannabinoids are exposed to heat or light, they decompose to decarboxylate molecules such as tetrahydrocannabinol (THC) or cannabidiol (CBD), correspondingly. THC corresponds to the psychoactive form while the acid forms and CBD are related to therapeutic properties, with antipsychotic effects. However, cannabinol (CBN) seems to be formed by the decomposition of its acid CBNA form (or its primary CBGA molecule) and even from THCA, which is an indicator of the thermal decomposition of cannabinoids (Figure 1). 

The cannabis market is changing with the presence of high-THC-content products, and new forms of cannabis and extract-based commercial products from the cannabis plant are becoming increasingly available. The cannabis herb contains about twice as much THC than a decade ago. In addition, in Europe, drug use or possession offences involving cannabis comprise 75% of the total drug law offences [6]. Currently, high-performance liquid chromatography (HPLC) is the most used analytical technique for cannabinoid determination [7,8]. However, the applications of non-destructive vibrational spectroscopy approaches based on infrared or Raman spectroscopy to analyze marijuana have increased in recent years [8]. Thus, Raman spectroscopy has been used to differentiate among cannabis (THCA-rich hemp), CBD-rich plants, and regular hemp in order to provide a tool for hemp farming/cultivation [9]. Portable Raman spectroscopy has been successfully used to probe the content of THCA in samples [10]. However, there are still open questions, such as whether Raman microscopy can identify marijuana and discriminate among different marijuana varieties. As a consequence, this work aimed to develop a chemometrics-assisted method based on Raman microscopy for differentiation between different marijuana varieties. 

## 2. Materials and Methods

### 2.1. Plant Material and Samples

Five marijuana (*Cannabis sativa* L.) samples were analyzed: four Sativa (Amnesia Hy-Pro, Amnesia Haze, Original Amnesia, and Y Griega) and one Indica (Black Domina) genetic varieties. These plant materials were donated by the Campoactivo grow shop (Alcalá de Henares, Spain), which produced those plants from their own selected seeds with the certification of the corresponding genotypes. All varieties studied are technically hybrids. However, they express strong Sativa phenotypes for the Sativa variety, with very high Sativa genotypes. Therefore, Amnesia Hy-Pro is mostly 60% Sativa, composed from a cross of Afghan and Neville’s Haze. Amnesia Haze is 70% Sativa and composed from a cross of staple strain Haze and several different worldwide landraces, including Thai, Hawaiian, and Afghani. Original Amnesia is 70% Sativa and it is the cross of a Haze and a Northern Lights. The Y Griega type is 80% Sativa composed from a cross between Amnesia Haze and Kali Mist. The Indica variety Black Domina is 95% Indica and its genetic makeup includes four powerful Indica varieties named Afghanistan, Canadian Ortega, Northern Lights, and Hash Plant. The marijuana samples were cultivated and prepared in the Grow Store, simulating a self-consumption with no special care in the drying procedure, which combined uncontrolled heating and room temperature. Those marijuana samples were received dried as a collection of buds and leaves and their seasoning of the cannabis plant materials. The plant material came in individually labelled small plastic hermetic containers. They were stored within locked dark cabinets in a temperature-controlled room to preserve them from any extreme heat and light sources. The samples considered in this study were analyzed after about one month following the plant harvesting and over three months from their arrival to the lab. 

Three standards were acquired (MilliporeSigma, aka Sigma Aldrich, Merck KGaA, St. Louis, MO, USA) for comparing the samples’ spectra: decarboxylated cannabinoids (−)-trans-Δ^9^-tetrahydrocannabinol (THC), cannabidiol (CBD), and cannabinol (CBN) (1.0 mg/mL in methanol). They were expected as major products from the dried cannabis plant.

### 2.2. Quantum Chemical Simulated Raman Spectra

Tetrahydrocannabinol acid (THCA), cannabidiol acid (CBDA), tetrahydrocannabinol (THC), and cannabidiol (CBD) were modeled by quantum chemical calculations using the Gaussian 16W and Gaussview 6 software packages [11]. The simulated Raman spectra were calculated from the optimized structures at the Def2TVZP level of calculation.

### 2.3. Raman Analysis and Spectra Acquisition

A DXR Raman confocal microscope spectrometer (Thermo Fisher Scientific, Waltham, USA) with a high-precision motorized stage allowed searching and focusing on the trichome heads (50 to 100 µm wide) of the cannabis samples. The excitation source was a diode-pumped solid-state (DPSS) He-Ne laser (nominal power: 20 to 25 mW) emitting at 532 nm. A CCD detector cooled by a thermoelectric cooler to minimize the electrical noise was used for recording the Raman scattering. 

The spectra of all the samples were collected using OMNIC32 Software (Thermo Fisher Scientific, Waltham, MA, USA) within the 400 to 4000 cm^−1^ Raman shift range. The spectrograph aperture was set to 25-µm-slit for the synthetic cannabinoids (reference compounds) and 50-µm-pinhole for the cannabis samples. The different adjustable aperture (slit/pinhole) permits better control of the amount of incoming laser radiation. Usually, the slit aperture results in a higher Raman signal than the pinhole aperture [12]. Therefore, to prevent the cannabis samples from burning, lower laser power (8 mW, at the sample) and a pinhole type-aperture were used.

**Figure 2:**
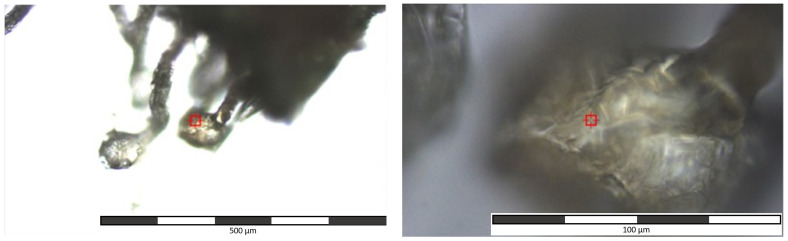
Optical microscope image of a trichome head used for collecting the spectra (**left**: 10× magnification, used for exploring the sample and finding representative trichomes; **right**: 50× magnification, used for collecting the spectra only from different spots on the selected trichomes).

The cannabis samples were intentionally measured without any sample pretreatment, and at different points in time. At the end, all the spectra were assembled for this study. Cannabis plant material comprises the collection of floral buds and leaves and their seasoning for marijuana. A small amount (a spatula tip) of every marijuana sample was laid over a microscopy slide covered with aluminum foil and observed with a 10× objective in the Raman microscope. The use of aluminum foil is a common Raman trick to avoid the fluorescence produced by the glass [12]. The focus was set on the trichome heads because they are the site of the cannabinoid production [13,14,15,16] and the THC content is expected to be the highest (15 to 25%) and consequently, there is a stronger spectral signal. This was especially important because uninformative spectra were obtained when randomly focusing and irradiating on any bulk marijuana sample. When a representative capitate-stalked or a bulbous trichome was encountered, the microscope objective was changed to 50× magnification to focus only on the trichome (drug-containing) head surface. Figure 2 shows an example of the trichome magnifications at 10× and 50×. The 10× magnification was only used for exploring the sample and finding representative trichomes. The 50× magnification was used for collecting the spectra only from the different spots on the chosen trichomes. This is practical, considering that at 50× magnification, the laser spot diameter irradiates an area of about 1.1 µm. In order to avoid burning the samples, three trichome heads of every marijuana sample were measured with the following conditions: 8 mW laser irradiation power (at the sample surface), 30 accumulations of 1 s acquisition time each. In total, 60 spectra were collected for every marijuana sample; that is, 20 spectra were always gathered from 20 different (randomly selected) points on every trichome’s head. The cannabinol standards were measured with the following conditions: 14 mW laser irradiation power (at the sample surface), 20 accumulations of 5 s acquisition time each.

### 2.4. Data Pre-Processing and Analysis

From the entire spectral range measured in this work (200 to 4000 cm^−1^), the spectral region of interest (ROI) considered for the chemometric analysis was 200–1800 cm^−1^. This range was selected because most of the marijuana’s Raman spectral fingerprint can be found around this region, which is important if the reader wants to perform a comparison. The whole raw ROI containing all the marijuana samples was pre-treated prior any data analysis. Data pre-processing is important to ensure that data are standardized and fully comparable. The pre-processing sequence was always performed verifying that neither the data nor the results were degraded; that is, negatively affected. The data were treated to correct the fluorescence background using a 5th-order polynomial baseline correction method built-in the Omnic software. This fluorescence may come from the chlorophylls, chlorophyllin, and analogous compounds, which are highly auto-fluorescent [16,17]. Then, wavenumber alignment was performed to compensate for the small offsets among and along the spectra. The wavenumber alignment consisted of shifting the wavenumber axis to align the spectral bands, to compensate for artificial shifts (i.e., artifacts) due to drifts in the laser emission wavelength or differences in the instrument optical alignment. This processing is performed since even very small shifts can impact the data analysis. The wavenumber alignment was performed using a custom interpolation function, choosing the first spectrum as the reference. Finally, the spectral intensity was normalized using the vector normalization procedure, which calculates the average intensity value for all (chosen) wavenumbers. Afterwards, the intensities are centered and divided by the length of the spectrum (as a vector); thus, the new spectrum vector has a length of 1. The data were pre-processed using the MALDIquant, baseline, and hyperSpec packages available within the R and RStudio v.3.3.2 and v1.0.136 software packages, respectively [18,19].

The multivariate analysis was carried out on the collected marijuana spectra for discriminating the Indica and Sativa genetics and the Sativa varieties whilst trying to explain their profile. The orthogonal partial least-squares discriminant analysis (OPLS-DA) was performed using SIMCA (Sartorius Stedim Biotech, Göttingen, Germany) software. OPLS-DA is a statistical modeling tool that combines orthogonal signal correction (OSC) and partial least-squares discriminant analysis (PLS-DA) [20,21,22,23]. It provides understanding about the separations among the experimental groups; in this case, based on data containing high-dimensional spectral measurements with multicollinear and noisy variables [20,22,23,24,25]. The default scaling for the X-block and Y-block in the default workset was set to the Pareto variance to compensate for any magnitude unbalance and/or variance. This enabled eliminating any weight due to the variables or observation magnitude. In the Pareto scaling, the base weight was computed as 1/sqrt(sdj), where sdj is the standard deviation of the variable j computed around the mean. The confidence level of the fitting parameters was set to 95%, and the significance level for Hotelling’s T^2^ was set to 0.05 [25]. The analysis was performed using a non-linear iterative partial least-squares (NIPALS) algorithm. The goodness of fitting, which included an uncertainty test, was assessed using the leave-one-out cross-validation (CV) method, where each observation is left out once during the cross-validation. Furthermore, the number of cross-validation rounds was kept to seven by default. This is the number of groups that one by one is left out of the modeling and repredicted during the cross-validation. Hence, because OPLS-DA is sensitive to the model complexity [25], and to avoid overfitting, a repeated 7-fold CV was set to estimate the relevant number of components in the OPLS models [26]. Nonetheless, in order to stay in the safe side of the model fitting and still have the optimal number of proper factors for generating a predictive model, less than five latent variables were used in all cases.

The OPLS-DA scatter plots allowed visualizing the discriminated groups along the resulting latent variables, as well as the corresponding experimental variables (wavelengths) that best described those groups’ behavior. After finding the models, the subsequent confusion matrices were used to report the fitting results, i.e., the performance of a classification model. Some results were compared using contribution plots, which are point-focused versions of the variable importance of the projection (VIP) plots. The contribution plots show why a specific point (or group of points) in a score or Hotelling’s T^2^ plot deviates from the average or from another point in the X-space. In other words, they show the weighted difference between the point’s data (scaled and centered as the dataset) and the average of the model. Here, the weights are by default derived from the underlying model. The plot shows one bar per active variable or term on the X-side. The vertical scale corresponds to the scaling of X, and since the X is Pareto-scaled in this workset, the vertical scale is given in terms of 1/sqrt(StdDevs) units. The largest bars denote which variables deviate most from the reference point (here the average), and the sign of the bar (up = plus/positive and down = minus/negative) designate in which direction the variables diverge.

## 3. Results and Discussion

The most utilized strategy to determine the cannabinoid profile of plant material and the quality of cannabis samples involves liquid–liquid extraction followed by high-performance liquid chromatography (HPLC). In addition, gas chromatography (GC) in conjunction with mass spectrometry (MS) or flame ionization detection (FID) can be employed for this purpose, but a derivatization step is essential if the acids need to be quantified [27]. Current methods of cannabinoid analysis present some disadvantages such as expensive equipment and alteration or destruction of the sample, and are time-consuming. An alternative approach is therefore here proposed for the fast and non-destructive cannabis material analysis consisting of the use of vibrational Raman spectroscopy.

### 3.1. Marijuana Spectral Characteristics

Since the capitate-stalked and bulbous trichomes of the marijuana contain high cannabinoid accumulation levels [13,14], they were the target structures to be located using a microscope as shown in Figure 2 [15].

**Figure 3:**
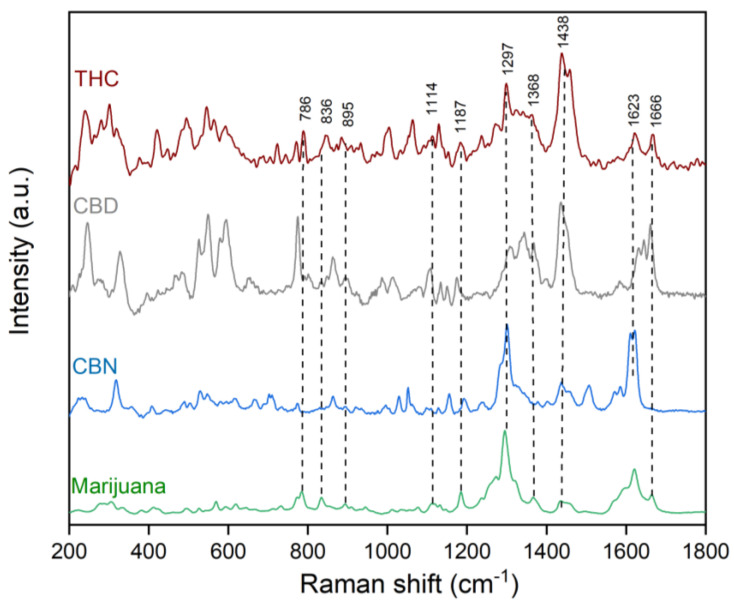
Experimental average Raman spectrum of marijuana and the main cannabinoids (THC, CBD, CBN) used as standards.

Figure 3 shows the average Raman spectrum of marijuana. This spectrum was obtained from the average spectra of the five studied marijuana types (Amnesia Haze, Amnesia Hy-Pro, Original Amnesia, Y Griega, and Black Domina). The average spectra of every marijuana type were visually very similar, with slight differences in the shifts and intensities of the Raman bands, which will be discussed later in the chemometric analysis. In addition, Figure S1 shows a normalized spectrum (at 1438 cm^−1^) of the marijuana and decarboxylated cannabinoids (THC, CBD and CBN). The signal at 1438 cm^−1^ is attributed to vibrations of the CH_2_ groups, which are usually present in many related structural biological compounds, in this case cannabinoids. This vibrational mode is in a free region, avoiding the influence of other bands, and it is suitable to use to normalize the spectra. 

Marijuana spectral bands were compared with those of the decarboxilated cannabinoid spectra (THC, CBD, CBN), since the laser was focused over the main production site of cannabinoids. The assignments of the Raman spectral features from the cannabis samples were performed within the 600–1800 cm^−1^ range, because this is where the more characteristic molecular vibration modes occur (molecular fingerprint). The spectral assignments were based on the information derived from the experimental and calculated Raman spectra of the cannabinoids and by comparison with previously reported data [9]. 

The Raman spectrum of marijuana was assigned considering mainly the three representative zones derived from the vibrational modes of the main types of groups in the chemical structure (C=C, C–C, CH_2_, CH_3_, C–O–C, and others) of its main constituents, the cannabinoids (Figure 1). The first zone at a higher wavenumber is related to the presence of the C=C double bonds. Indeed, the bands at 1666 cm^−1^, 1623 cm^−1^, and the shoulders at 1600 and 1570 cm^−1^ are in the C=C stretching region and were assigned with confidence to these cannabinoid modes. Figure S1 shows the spectra normalized to 1438 cm^−1^, which roughly lets us appreciate a compound’s contribution to the marijuana spectrum. As can be seen, the overlapped spectra of the THC, CBD, and CBN molecules suggest the presence of more related-structure molecules. It seems that the precursor acid forms could also be present and contributed to this broad band. Figure S2 shows the theoretical calculated Raman spectra of both acid and decarboxylated cannabinoid molecules. It allows visualizing the very similar position of the C=C stretching modes; therefore, it is appropriate to also assign the contributions of the acid form molecules to this spectral region. Further, a related-structures contribution cannot be ruled out considering the tens of marijuana cannabinoids elucidated previously.

The second representative region of the marijuana Raman spectrum was located between 1500 and 1100 cm^−1^, which is characterized by the different types of CH_2_ bending signals. According to the simulated vibrations of THC, CBD, and their acid precursors, the CH_2_ scissor bending was expected to appear at higher wavenumbers. Hence, the bands at around 1438 cm^−1^ were assigned to these marijuana cannabinoid modes. Then, the band at 1297 cm^−1^ and those closer were attributed to the CH_2_ bending of cannabinoids. This was predicted by quantum chemical calculations, which agree with previous reports regarding related structures. Redshifting was observed within other lower intense bands at 1187 and 1114 cm^−1^ in the marijuana spectrum. This agrees with the region of the CH_2_ twist bending modes, in this case attributable to the main components of the trichomes; the cannabinoids. 

The lower wavenumber region of the marijuana spectrum (1100 to 600 cm^−1^) was less intense. However, some well-defined bands were observed at around 1079 cm^−1^, which were attributed to the C-C stretch vibrations of the alkyl groups belonging to the expected cannabinoids. Likewise, the bands at 836 and 786 cm^−1^ agreed with the CH_3_ and CH_2_ rocking modes of the same molecules in the trichomes of marijuana. 

It must be noted that the marijuana’s Raman spectrum is representative of its main components. Cannabinoids have remarkably similar structures characterized by alkyl, aromatic, and heterocycle groups. Furthermore, the similarities in their Raman spectra are also high, which result in complex and unambiguous assignment of the whole spectra. Even more, lignin, cellulose, pectin, and carotenoids could also be present because the trichome is a vegetable tissue. For example, the band at 1185 cm^−1^ could also result from the contribution of the C−O−H stretching next to the aromatic ring and the CH bending, assigned to xylan. Likewise, the band at 1114 cm^−1^ could be assigned to the vibrational modes of the C−O−C symmetric and C−OH bending of cellulose [9]. In this regard, a general description of the marijuana Raman spectrum was present in the previous paragraphs, as it may be more suitable and useful to understand the varied classification results of the marijuana Raman spectra when using chemometric methods. 

### 3.2. Marijuana Classification

**Figure 4:**
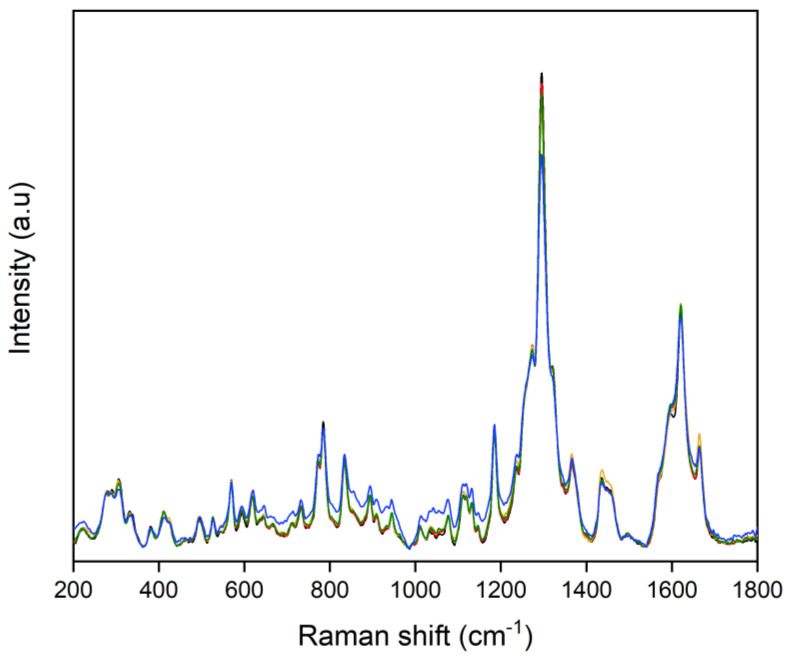
Raman data set highlighting the similarities and differences among the average spectra of the Indica (Black Domina, in black) and Sativa varieties (Amnesia Haze, in red; Amnesia Hy-Pro, in gold, Original Amnesia, in blue; and Y Griega, in green).

Figure 4 shows the comparison of the average spectra belonging to the Indica and Sativa genetic variants in the 200–1800 cm^−1^ spectral range. In general, both spectra are visually very similar with just a few differences in the intensity and peak features along the spectra.

Some of the weak spectral differences among the samples are visually observable especially in the 800–1200 cm^−1^ range. Moreover, the visual intensity relation between the bands at 1300 and 1600 cm^−1^ looks different amid the Indica and Sativa samples. Nonetheless, a classification OPLS-DA method was explored to test whether the different cannabis varieties could be differentiated.

**Figure 5:**
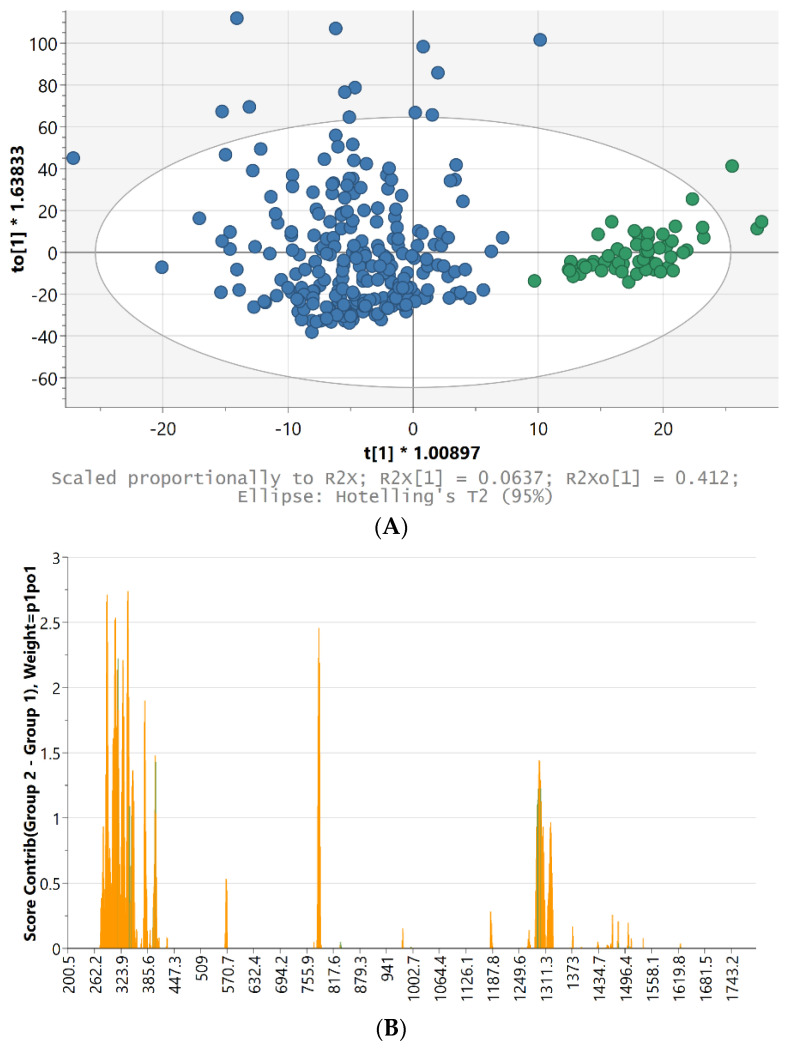
(**A**) 2D score scatter plot from the OPLS-DA model applied to the Indica (green) and Sativa (blue) marijuana Raman spectra. (**B**) Contribution plot for the Indica against Sativa marijuana samples. The variables (wavelengths) colored in orange contribute the most to make the Indica class different from the Sativa class.

Figure 5A shows the 2D score scatter plot from the OPLS-DA model, which differentiates the class within the Indica and Sativa marijuana spectra. This chemometric technique allowed distinguishing the two genetic variants. The vertical to[1] orthogonal direction (axis) expresses within-class variability, which is unrelated to the question of discriminating between the two classes, but it is still important for the total understanding of the problem.

The 7-fold (groups) CV autofit algorithm suggested one predictive t[1] and eight orthogonal to[1–8] variables. However, as can be seen, the separation of the genus was already clear using only one predictive t[1] and one orthogonal to[1] variable. The R2X[1] parameter indicated that about 6.4% of the X variation was modeled by the predictive t[1] component. Likewise, R2Xo[1] showed that 41.2% of the X variation was modeled by the orthogonal to[1] component. Moreover, a total of 62.1% of the X variation was modeled by the four orthogonal to[1–4] components, which rendered a rather good 68.5% explanation of the separation. The outliers outside the 95% confidence Hotelling’s T^2^ ellipse were not excluded from the analysis because in spite of their condition, in perspective, they did not affect the overall separation results.

Figure 5B shows the top (positively marked) part of the contribution plot for the Indica versus Sativa samples. This plot shows the variables (wavelengths) that contribute to make the Indica class so different from the Sativa class. The variables colored in orange contribute the most because the original variables are outside the ±3 standard deviation (StdDev) limits for the corresponding observations. Hence, the following band and shoulder region results were quite important for the Indica discrimination: 346–354 and 359–362 cm^−1^. Some other important bands and band regions were 283, 290–293, 309–312, 317, 330, 339–341, 380, 404, 570, 785, 1296–1300, and 1324 cm^−1^.

A confusion matrix is a specific table layout that allows visualizing the performance of an algorithm, which represents the statistic classification accuracy, specificity, and sensitivity. In this case, Table 1 shows the confusion matrix for a 7-fold cross-validation classification procedure on the marijuana genetic dataset. Its 100% high classification accuracy indicates that it is possible to distinguish the marijuana genetics from Raman spectra taken from marijuana trichomes.

**Figure 6:**
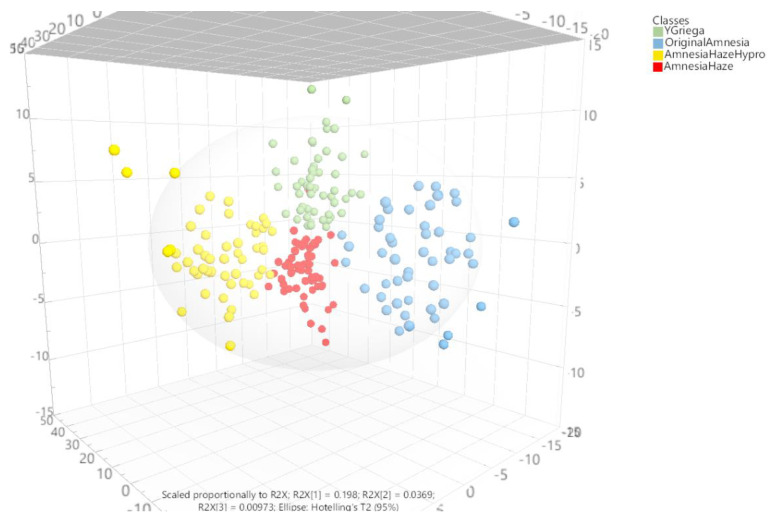
3D score scatter plot from the OPLS-DA model applied to the Sativa marijuana Raman spectra in order to differentiate the sample types. Supplementary Figure S3 shows the individual 2D representations of these 3D planes.

Given these results and in order to discriminate between the Sativa sample (variety/type) classes if possible, a new OPLS-DA model was created without the Indica samples. Although the separation was rather good considering all spectra, a few strong outliers were removed from the analysis as they were influencing the model too much. Figure 6 shows the separation of the sample types by OPLS-DA after auto-fitting three predictive t[1–3] and eight orthogonal to[1–8] variables (latent variables or components). On the one hand, the three predictive t[1–3] components accounted for 24.4% of the total variability, from which 19.8, 23.4, and 24.4% of the X variation was modeled by the t[1–3] components. On the other hand, the eight orthogonal components to[1–8] explained 54.8% of the X variation. In other words, the entire model explained 79.3% of the sample variability, which represents a rather good discrimination model. The remaining outliers outside the 95% confidence Hotelling’s T2 ellipse were not excluded from the analysis for the sake of showing that despite their nature, they did not significantly affect the overall discrimination results.

The first component, t[1], was able to discriminate Original Amnesia from the other samples, especially YGriega and AmnesiaHaze. The second component, t[2], was good for separating Amnesia Haze Hypro from the other sample types, especially YGriega and AmnesiaHaze. The third component, t[3], was rather good at separating YGriega from AmnesiaHaze. However, there were some samples that were confounded in the zero zone of each OPLS-DA component; the full discrimination results are better appreciated in a 3D perspective scatter plot (Figure 6).

Table 2 shows the confusion matrix for a 7-fold cross validation classification of the Sativa marijuana types. Its high classification accuracy of 100% indicates that it was possible to distinguish the various Sativa marijuana types considering their Raman spectra.

Various contribution plots were created in order to find the bands responsible for such a separation among the Sativa genus (Figure S4). The Original Amnesia class (Figure S4A) is visually rather different from the other Sativa types that can be distinguished in a group. According to its corresponding contribution plot, most of its bands influenced its differentiation. The most important (the tallest) bands in the contribution plot corresponded to small bands and shoulders along the Original Amnesia spectra but mainly in the central spectral region.

Although the spectra of the Amnesia Haze Hypro class (Figure S4B) visually does not stand out significantly from the other classes, it has a few bands and shoulders that differ in the extremes of the spectral range. The most important bands and shoulder regions for differentiating the Amnesia Haze Hypro class were at about 1360, 1440 (probably corresponding to the bending vibration mode of the aliphatic CH_2_ and CH_3_ of the cannabinoids), 1634, and 1666 cm^−1^ (C=C stretching vibrational mode of the cannabinoids).

The following bands were quite important for the complete YGriega differentiation (Figure S4C) among the other classes: 1313, 1386 (those in the CH_2_ bending vibration of the cannabinoids’ aliphatic region), 1570, and 1595 cm^−1^. For the Amnesia Haze class (Figure S4D), the band and shoulder regions important for its differentiation were 981, 1275, 1495, and 1595 cm^−1^. They corresponded to some initial bands and shoulders plus the largest bands and shoulders in their spectral range. It can be noted that YGriega and Amnesia Haze classes had strong coincidences at the 397, 411, 985, 1258, 1293, 1300, 1313, 1318, 1495, 1591, and 1595 cm^−1^ bands.

## 4. Conclusions

Raman spectroscopy parameters need to be properly selected to measure marijuana trichomes. Thus, a precise focusing over the trichome zone and low laser intensity were necessary to obtain good Raman spectra while avoiding sample damage.

The Raman spectra of the Δ^9^-tetrahydrocannabinol (THC), cannabidiol (CBD), and cannabinol (CBN) standard cannabinoids were compared with the Raman spectra of five different types of marijuana: four Sativa varieties (Amnesia Haze, Amnesia Hy-Pro, Original Amnesia, and Y Griega) and one Indica variety (Black Domina). The results show the presence of several common spectral bands, which are useful for marijuana characterization. These findings were supported by quantum chemical simulated Raman spectra of the cannabinoid acid-forms (tetrahydrocannabinol acid (THCA), cannabidiol acid (CBDA)) and decarboxylated cannabinoids (THC, CBD, and CBN).

A chemometics-assisted method based on Raman microscopy and an OPLS-DA model offered good classification and discrimination of the different marijuana genetics and varieties, which opens interesting perspectives in the forensic field.

## Figures and Tables

**Table 1:** Confusion matrix for the marijuana genetics classification.

Predicted				
**Actual**		**Indica**	**Sativa**	**Sum**
	**Sativa**	238	1	239
	**Indica**	0	60	60
	**Sum**	238	61	299

Fisher’s probability was 0 and is satisfied when *p* < 0.05 for a 95% confidence level. For the calculation of Fisher’s probability, all probabilities more extreme than the observed pattern are computed and summed to give the probability of the table occurring by chance.

**Table 2:** Confusion matrix for the Sativa marijuana type classification.

Predicted						
**Actual**		**YGriega**	**OriginalAmnesia**	**AmnesiaHazeHypro**	**AmnesiaHaze**	**Sum**
	**YGriega**	54	0	0	0	54
	**OriginalAmnesia**	0	49	0	0	49
	**AmnesiaHazeHypro**	0	0	53	0	53
	**AmnesiaHaze**	1	0	0	58	59
	**Sum**	55	49	53	58	215

Fisher’s probability; that is, the probability that this table may occur by chance was 0.

## Data Availability

Not applicable.

## Supplementary Materials

The following supporting information can be downloaded at: https://www.mdpi.com/article/10.3390/toxics10030115/s1, Figure S1: Normalized Raman spectra (at 1438 cm^−1^) of those in Figure 3; Figure S2: Experimental Raman spectrum from the marijuana trichomes and quantum chemical simulated spectra for the main expected marijuana cannabinoids at the Def2-TZVP calculation model; Figure S3: 2D Scores Scatter plots representing the three planes forming the 3D Scores Scatter plot from the OPLS-DA model (Figure 6) applied to the Sativa marijuana Raman spectra in order to differentiate the sample type; Figure S4: Contribution (loadings-focused) plots for the various Sativa samples analysed in this work. (**A**) Original Amnesia (top part) compared to the rest of the samples. (**B**) Amnesia Haze Hypro (top part) compared to the rest of the samples. (**C**) YGriega (top part) compared to the rest of the samples. (**D**) Amnesia Haze (top part) compared to the rest of the samples.
