# Supplementary material for: Classification of Various Marijuana Varieties by Raman Microscopy and Chemometrics
Source: Toxics. 2022 Feb 28;10(3):115. doi: 10.3390/toxics10030115 (PMC8948958; doi:10.3390/toxics10030115)
Supplement: Supplementary file 1 [file toxics-10-00115-s001.zip › toxics-1585478-supplementary.pdf]

toxics MDPI

Supplementary Materials: Classification of Various Marijuana Varieties by Raman Microscopy and Chemometrics

Luis Ramos-Guerrero, Gemma Montalvo, Marzia Cosmi, Carmen García-Ruiz and Fernando E. Ortega-Ojeda

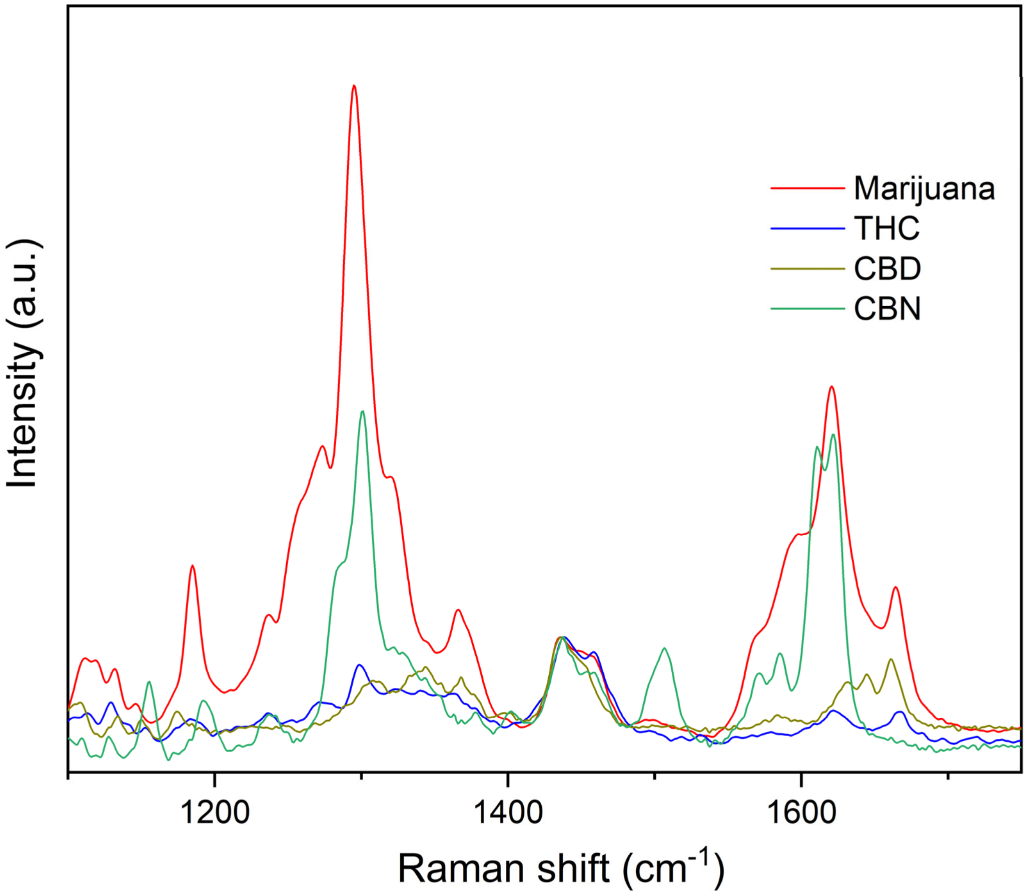

Figure S1. Normalized Raman spectra (at 1438 cm^{-1}) of those in Figure 3.

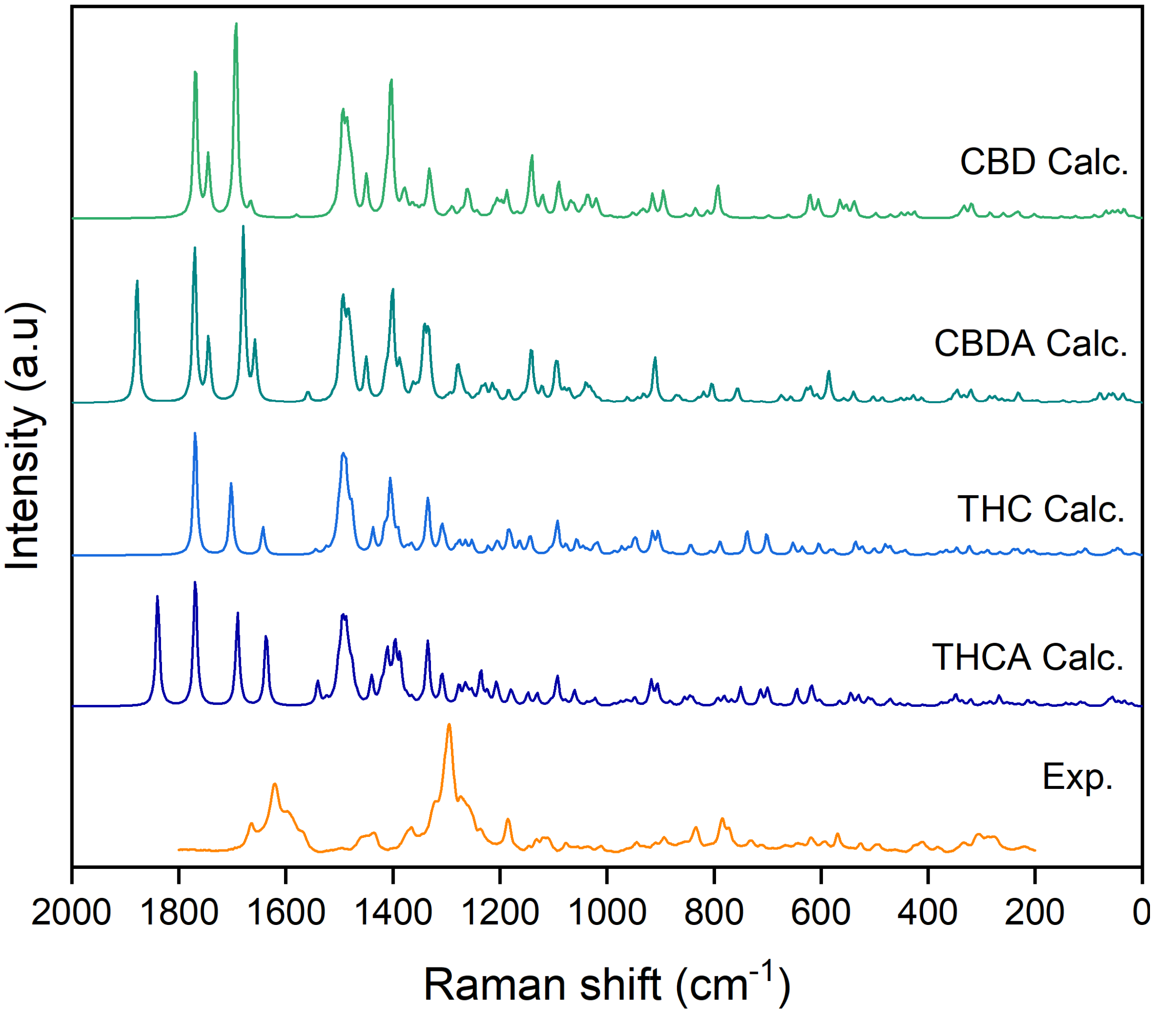

Figure S2. Experimental Raman spectrum from the marijuana trichomes and quantum chemical simulated spectra for the main expected marijuana cannabinoids at the Def2-TZVP calculation model.

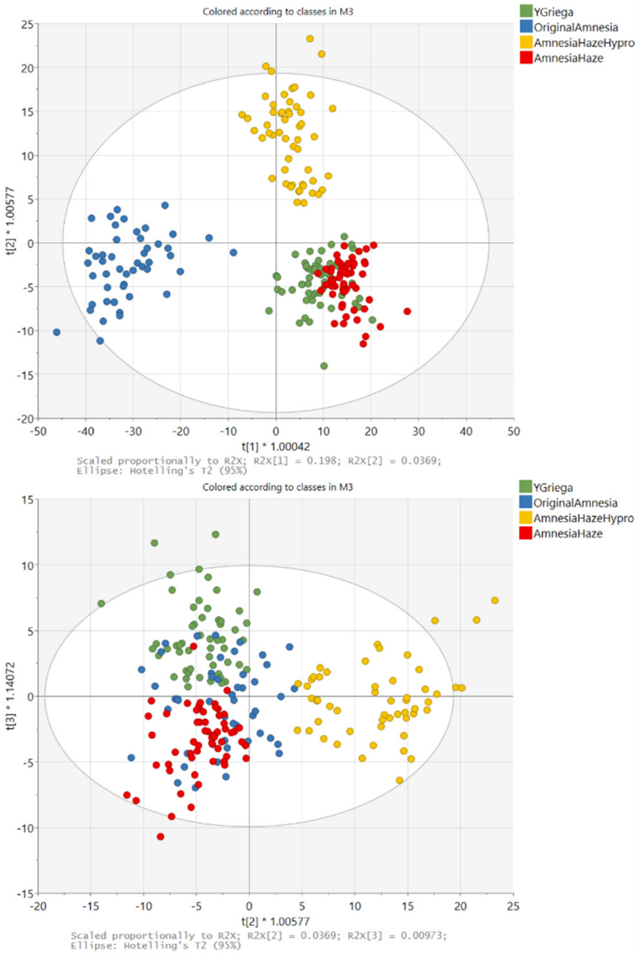

Figure S3. 2D Scores Scatter plots representing the three planes forming the 3D Scores Scatter plot from the OPLS-DA model (Figure 6) applied to the Sativa marijuana Raman spectra in order to differentiate the sample type.

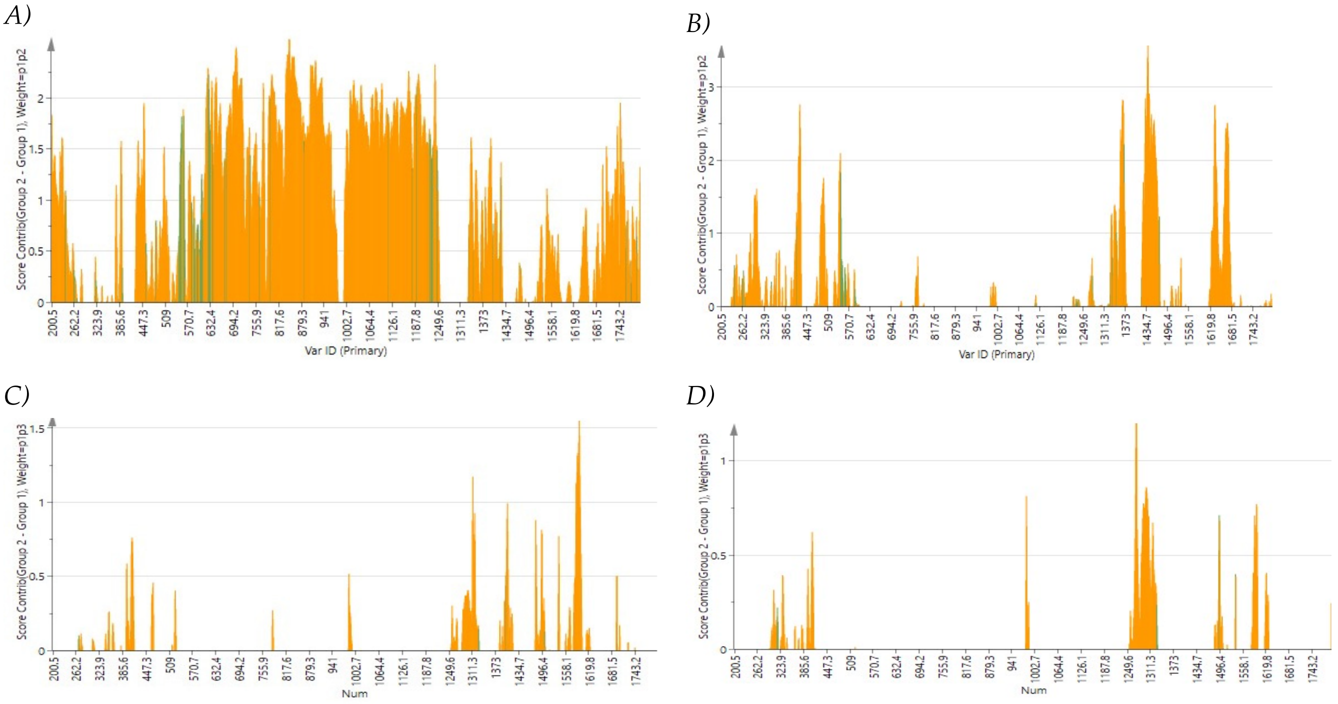

Figure S4. Contribution (loadings-focused) plots for the various Sativa samples analysed in this work. (**A**) Original Amnesia (top part) compared to the rest of the samples. (**B**) Amnesia Haze Hypro (top part) compared to the rest of the

samples. (**C**) YGriega (top part) compared to the rest of the samples. (**D**) Amnesia Haze (top part) compared to the rest of the samples.
